# Hallmarks of Testicular Aging: The Challenge of Anti-Inflammatory and Antioxidant Therapies Using Natural and/or Pharmacological Compounds to Improve the Physiopathological Status of the Aged Male Gonad

**DOI:** 10.3390/cells10113114

**Published:** 2021-11-10

**Authors:** María Eugenia Matzkin, Ricardo Saúl Calandra, Soledad Paola Rossi, Andrzej Bartke, Mónica Beatriz Frungieri

**Affiliations:** 1 Instituto de Biología y Medicina Experimental, CONICET, Ciudad de Buenos Aires C1428ADN, Argentina; rscalandra@hotmail.com (R.S.C.); rossi@dna.uba.ar (S.P.R.); mfrungieri@dna.uba.ar (M.B.F.); 2 Departamento de Bioquímica Humana, Cátedra I, Facultad de Medicina, Universidad de Buenos Aires, Ciudad de Buenos Aires C1121ABG, Argentina; 3 Geriatrics Research, Department of Internal Medicine, School of Medicine, Southern Illinois University, Springfield, IL 62794, USA; abartke@siumed.edu; 4 Cátedra de Química, Ciclo Básico Común, Universidad de Buenos Aires, Ciudad de Buenos Aires C1405CAE, Argentina

**Keywords:** aging, testis, inflammation, oxidative stress, apoptosis, anti-aging

## Abstract

The evolutionary theory of aging supports a trade-off relationship between reproduction and aging. Aging of the male reproductive system primarily affects the testes, leading to a decrease in the levels of sexual hormones, alterations in sperm quality and production, and a decline in fertility that does not necessarily involve a complete cessation of spermatogenesis. Inflammation, oxidation, and apoptosis are events considered as predictors of pathogenesis and the development of age-related diseases that are frequently observed in aged testes. Although the molecular mechanisms are still poorly understood, accumulating evidence points toward pro-inflammatory molecules and reactive oxygen species as primary contributing factors for testicular aging. However, the real impact of aging-related testicular alterations on fertility, reproductive health, and life span is far from being fully revealed. This work discusses the current knowledge on the impact of aging in the testis, particularly of aging-related dysregulated inflammation and oxidative damage on the functioning of its different cell populations. More interestingly, this review covers the potential benefits of anti-aging interventions and therapies using either pharmacological compounds (such as non-selective non-steroidal anti-inflammatory medication) or more natural alternatives (such as various nutraceuticals or even probiotics) that exhibit anti-inflammatory, antioxidant, and anti-apoptotic properties. Some of these are currently being investigated or are already in clinical use to delay or prevent testicular aging.

## 1. Introduction

**Figure 1:**
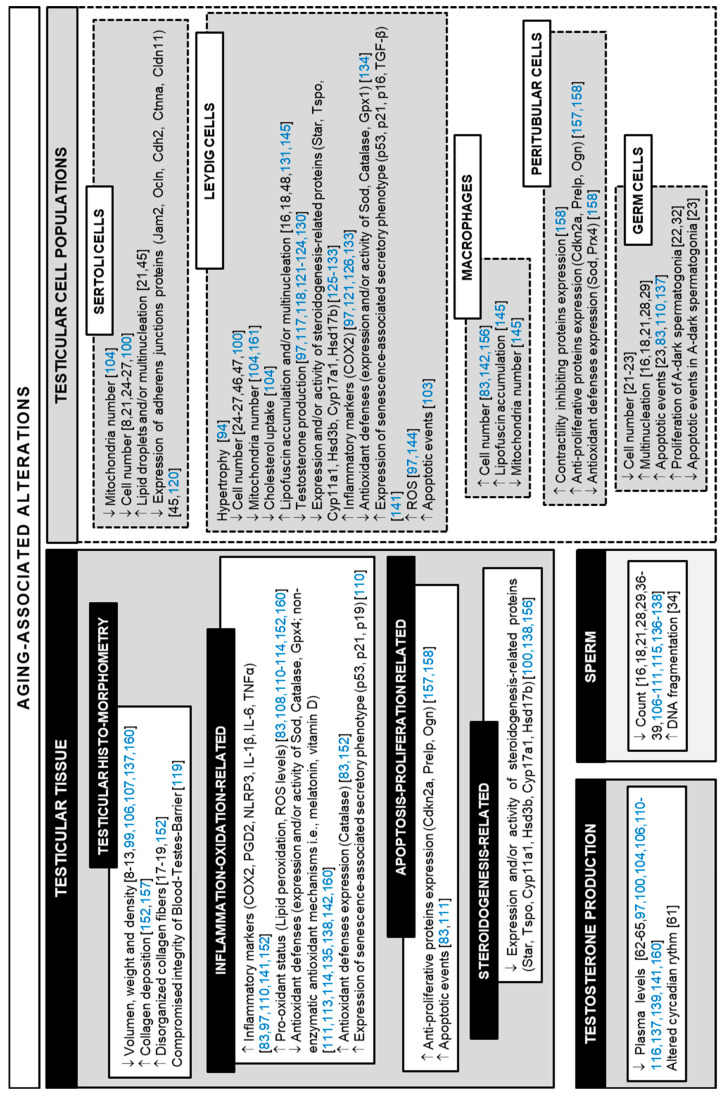
Primary alterations reported in the testis of aging men and in aged experimental animals. Corresponding references are listed between brackets (black, data on human samples; blue, data on animal models).

Aging is accompanied by numerous changes in the function of the endocrine system. Many of these changes are very pronounced, readily detectable, and have been thoroughly documented [1]. Recent studies have focused more on the aging-related dysregulation observed in the inflammatory and oxidative status of the testis [2,3], which is in line with the commonly accepted oxidation–inflammation theory of aging described for several extra-gonadal tissues [4]. In general terms, this theory suggests that the aging process is linked to chronic oxidative stress, which affects all cells of the organism, especially immune cells. Thus, being unable to preserve their redox balance, cells would suffer functional losses incompatible with suitable preservation of homeostasis that drives the over-expression or over-production of inflammatory markers. Overall, literature on this topic seems to support the idea that a pro-inflammatory and pro-oxidant microenvironment is characteristic of aged testes. Thus, in this article, we will provide an overview of the impact of aging in the testis with emphasis on recent findings on the relevance of the oxidation–inflammation theory of aging (Figure 1).

As men age, testicular function declines gradually, affecting not only their fertilizing capacity but also their overall health status and quality of life [3,5,6,7]. However, consensus on the importance of the study of the reproductive aging process, as well as the communication of such a topic to the general public, have been, thus far, very limited. There is controversy regarding the form in which testicular aging should be diagnosed, treated, or even denominated; many go as far as to question its clinical entity. Although aging constitutes a universal, multi-factorial, progressive, and irreversible process, many attempts have been made to delay or prevent aging in general. Studies on testicular aging are becoming more and more relevant given that, in modern societies, paternal age has increased. Furthermore, age-related decline in testicular function also impacts overall health status [3,5]. Importantly, although regional, ethnic, and socio-economic differences should be considered, life expectancy of human males worldwide is approximately 80 years, and it often surpasses the age of 90 years. Sexual activity continues into the eighth decade of life for many older men, and sexuality is an important emotional and physical component that actually ‘defines’ the quality of life in aging men [5]. 

Several studies, conducted primarily in animal models, have shown that the use of specific pharmacological compounds or more natural alternatives with known antioxidant and anti-inflammatory properties have a beneficial effect on the inflammatory and oxidative status of the aged testis. This review will discuss the current knowledge of such compounds (some of them, commonly used for the treatment of different pathologies) and the possible perspectives of employing them as anti-aging therapies in the testis.

## 2. Testicular Aging

### 2.1. General Aspects of Age-Dependent Alterations in the Human Testis

The aging process of the testis is quite relative, as there is no precise clinical symptom for the decline in testicular function. Although morphological studies of the testis of elderly men reflect pronounced individual variations, testicular morphology is, in general terms, profoundly altered during aging. Most studies performed in human tissue have shown decreased testicular volume, weight, and density [8,9,10,11,12,13], reduction of testicular perfusion [14], occlusion and thickening of blood vessels [14,15], increased tunica albuginea weight and thickness [8,11,16], thickening of basement membrane and an intensely collagenized and thickened tunica propria [17,18,19].

Age-related changes in testicular volume are essentially prominent in the seminiferous tubules [20]. The decrease in length and diameter that has been reported for aged seminiferous tubules [10,20] is the consequence of the loss of both germ cells [21,22,23] and Sertoli cells [8,21,24,25,26,27]. The most frequent histological pattern of the aging testis is a mosaic of different seminiferous tubule lesions, which vary from tubules with complete, although reduced, spermatogenesis, to completely sclerosed tubules [10,21]. Altogether, these reports indicate that abnormal histological structure and impaired spermatogenesis leading to germ cell loss are always present in the aging human testis [23]. On average, the loss of germ cells begins with the spermatids, but gradually affects the earlier stages of germ cell line. Hence, tubules with maturation arrest at the level of the spermatocytes or spermatogonia can be observed in aged testes [21,22,23]. In the meantime, in tubules with complete spermatogenesis, numerous morphological abnormalities in germ cells have been reported, including multinucleation originated from cell–cell fusion [16,18,21,28,29]. Differentiating germ cells only exist for the duration of one spermatogenic cycle, which, in men, is completed within 72 days [30,31]. Thus, only spermatogonial stem cells can be suspected to be really exposed to age-dependent processes. Very interesting studies performed by Pohl et al. [32] in testis from men with normal spermatogenesis revealed age-dependent, highly specific processes taking place in aging germ cells that are clearly distinct from somatic aging. In these studies, the authors propose aging-associated changes in the spermatogonial dynamics, in which elevated numbers of proliferating A-dark spermatogonia result in a loss of quiescence of these undifferentiated cell populations, in an effort to repopulate the testis. This decreases spermatogenic efficiency and leads to stem cell exhaustion and, possibly, to accumulating DNA replication errors, given the already reported decreased efficiency of DNA repair mechanisms in the aging testis revised by [33]. However, findings about DNA damage and apoptosis in the human testis are inconclusive and conflicting. Both decreased apoptosis in spermatogonia [22] and increased germ cell apoptosis [23] have been reported in aging men. Because human reproductive aging has been studied mainly without considering confounding factors like infertility or aging-related morbidities, both of which impact spermatogenesis, very few reports can actually claim that their results are solely aging-related changes, especially when it comes to gamete production. In this regard, Pohl et al. [34] have recently reviewed the literature focusing on data from healthy men or men with normal spermatogenesis, revealing an increase in sperm DNA fragmentation, an increase in telomere length, and changes in DNA methylation patterns in aging sperm. 

It is well established that as men age, sperm production and semen quality become altered. However, even though population-based studies frequently have a large sample size, they generally do not screen the subjects for health problems that might affect semen quality. For example, reproductive disorders such as hypogonadism or prostatic hyperplasia could affect semen and sperm parameters [35]. Therefore, careful consideration is necessary when trying to consider such alterations as solely related to the physiological aging process. Nevertheless, several studies (including meta-analyses and studies where only healthy men had been enrolled) have reported on significant decreases in semen volume, sperm motility, and normal sperm morphology in older men, while sperm count tended to decrease and sperm concentration either decreased or remained unchanged [36,37,38,39]. In addition, the major reason for diminished semen quality seems to involve the accumulation of reactive oxygen species (ROS) that accompanies aging [40,41].

ROS consist of both free radical and non-free radical oxygen-including molecules such as superoxide anion (O2●^−^), proxyl radical (●ROO), hydroxyl radical (●OH), singlet oxygen (^1^O_2_), and hydrogen peroxide (H_2_O_2_). To combat ROS, mammalian cells have naturally occurring antioxidant capacity, including enzymatic factors (e.g., superoxide dismutase 1 and 2, catalase, glutathione peroxidase, and peroxiredoxins,) as well as non-enzymatic factors (namely, glutathione, n-acetylcysteine, vitamins E, A, and C, coenzyme Q10, carnitines, myo-inositol, lycopene, selenium, zinc, and copper). Low levels of ROS are necessary for normal sperm function (e.g., capacitation, hyperactivation, acrosomal reaction, and fertilization) [42,43]. High levels of ROS can overcome the naturally occurring antioxidant capacity in the seminal plasma and cause oxidative stress. Human spermatozoa membranes are particularly rich in unsaturated fatty acids and are very sensitive to ROS-induced lipid peroxidation [44]. 

Because ROS and the resulting oxidative stress are intimately related to apoptosis, the increased apoptotic events that have been described in the human testis during aging [23,34] could also be, at least in part, explained by the accumulating ROS in aged testes. As degenerating abnormal cells that arise upon aging, (such as germ cells) are phagocytosed by Sertoli cells, an accumulation of lipid droplets in the Sertoli cell cytoplasm ensues [21,45]. However, this is not the only change that has been reported in Sertoli cells during aging. Multiple morphological abnormalities have been described for this cell population, including multinucleation [21,45], mitochondrial metaplasia [18], and reduction of cell junctions [45], along with a marked reduction in number [8,21,24,25,26,27]. 

While changes in testicular volume are prominent in the seminiferous tubules, the volume occupied by the testicular interstitium remains unchanged [10]. There have been contradictory findings in the few studies examining Leydig cell population number during aging. Some studies have described no change in Leydig cell number [21,24,26], while others have reported a reduction in both Leydig and interstitial cell number [46,47]. Recently, Mularoni et al. [27] have reported that increasing age was negatively associated with Leydig cell number. Apart from cell number, other primary morphological alterations of Leydig cells that are normally observed in aged testes are cellular atrophy, abundant intranuclear inclusions, multinucleation, a decrease in the quantity of smooth endoplasmic reticulum and mitochondria, the tendency to form small clusters, and an increase in lipofuscins and lipids [16,18,48]. 

Lipofuscin is the term given to yellow-brown pigment granules composed of lipid- and protein-containing residues of lysosomal digestion that, upon plasma membrane, mitochondrial or lysosomal damage, can become oxidized. Lipofuscin accumulates in post-mitotic cells due to its highly complex cross-linked structure that is not amenable to degradation. High lipofuscin load has been implicated in cell aging [49,50,51,52]. Lipofuscin accumulation has been suggested to negatively influence cellular functions by inhibiting the proteasome, hampering autophagy and lysosomal degradation, thus contributing to ROS generation. Defective cytosolic protein degradation, in turn, leads to the decreased degradation of pro-apoptotic proteins (including c-jun, Bax, and p27), triggering the initiation of the apoptotic cascade [53].

According to the oxidation–inflammation theory of aging, there is an underlying interdependence between oxidative stress and the occurrence of inflammatory processes in the age-related impairment of the functions of organisms. In fact, inflammation has been reported to be strongly associated with aging. The term “inflammaging”, which implies the occurrence of a global reduction in the capability to cope with a variety of stressors and a concomitant progressive increase in the pro-inflammatory status [54], is presently used to describe the up-regulation of the inflammatory response that takes place with advancing age. Increased circulating levels of pro-inflammatory cytokines (e.g., IL-1β IL-6, IL-18, TNF-α) and activation of certain immune cells have been reported in older people [55,56,57]. However, to our knowledge, there are no reports on testicular expression levels of inflammatory markers in aged human testes. Such analysis has only been conducted in experimental animals, which we will discuss later on.

Autophagy is an evolutionarily conserved process for cellular homeostasis through the degradation of long-lived proteins and functionally redundant or damaged intracellular organelles in lysosomes. Reduced autophagy is associated with accelerated aging, aging-related frailty and diseases, while enhanced autophagy partially protects cells from the natural aging process [58] and delays aging-related frailties [59]. New data suggest that elevated autophagy may suppress the activation of the NLRP3 inflammasome and reduce NLRP3-related inflammation [60]. In this context, protective effects of autophagy in inflammatory diseases associated with NLRP3 inflammasome have been reported, including gouty arthritis and familial Mediterranean fever. In contrast, autophagy dysfunction can lead to diseases featuring excessive activation of NLRP3 inflammasome and hyper-inflammation [60]. Inflammasome signaling pathways also regulate the autophagic process for maintaining the balance between defensive inflammatory responses and the prevention of excessive and detrimental inflammation [60]. Whether autophagic dysfunction is involved in aging-related testicular alterations observed in men needs to be further addressed.

Perhaps the most significant change in male reproductive aging that is directly associated to aging-dependent alterations in the human testis has to do with the decline in its endocrine function, namely the decline in testosterone production. In this context, a study from Bremner et al. [61] have demonstrated that not only there was a clear decrease in serum testosterone levels in healthy old men compared to those of young men, but that the early morning rise in testosterone levels characteristic of young men was not present in old age. Previous reports using single blood samples obtained in the morning (8–10 a.m.) have also shown a decrease in plasma testosterone levels in aged men compared to those found in young men [62,63,64]. Moreover, in recent years, longitudinal studies have reported greater annualized declines in plasma testosterone and DHT in older men (transitioning from 8th to 9th decades) over a 5-year follow-up when compared to younger men [65]. In contrast, a study from the 80’s comparing serum testosterone levels in normal aging men and normal young men had failed to show significant differences [66]. A plausible explanation for such discrepancies could be associated with the time chosen for sample collection and the impaired circadian rhythm in serum testosterone levels in aging men. Concomitant with reduced testosterone synthesis, elderly men, who are otherwise healthy, have increased serum levels of hormones that stimulate testosterone synthesis, such as LH and FSH. In addition, testosterone metabolites like estradiol, as well as inhibin, which is a factor involved in the negative feedback loop controlling testosterone synthesis, are significantly lowered [67,68]. The androgen deficiency that occurs with aging is referred to as late onset hypogonadism and is characterized by many disorders including low libido, erectile dysfunction, infertility, gynecomastia, hot flashes, low energy, sleep disturbance, depressed mood, impaired cognition, osteoporosis, and loss of muscle mass or increased body mass index [69]. Altogether, these symptoms constitute an impairment of health and quality of life. Consequently, elucidating the underlying mechanisms of testicular aging and identifying interventions that might slow down or postpone this process is a significant unmet health issue.

### 2.2. Animal Models for the Study of Testicular Aging: What We Know So Far

Given the poor, and sometimes ethically impeded, access to fresh, disease-free testicular tissue from old men, it has been rather difficult to obtain data on isolated testicular cell population physiology and the corresponding underlying regulatory mechanisms involved in their proposed impaired function during aging. Although there is conflicting evidence about the extent to which aging is a process that is similar across all organisms or particular to each species [70], the integrative understanding of aging implies that a diversity of model organisms will be essential to achieve a full understanding of the aging process. In fact, model organisms have been vital to the common goal of identifying and understanding the molecular, cellular, and environmental factors affecting longevity and improving healthspan. While many different non-human organisms have been used to explore the aging process (e.g., yeast, roundworms, and fruit flies), rodents (such as mice and rats) are routinely the models of choice. From the perspective of aging biology, several life characteristics make rodents an extremely appealing group for comparative studies, from the diversity in their maximum lifespans, to the many similarities they share with aging in humans. Furthermore, their short lifespans (compared to humans) and the ability to control environmental exposure create opportunities for regulatory up-regulation of lifespan. 

Studies in human populations have explored longevity candidate genes; a small but growing number of gene variants contributing to known longevity mechanisms has been established, including genes related to stress resistance, metabolism, and cellular division. In addition, over the last few decades, the relative ease of manipulating genes of interest has allowed for the development of organisms with specific genetic mutations to address the mechanistic aspects of longevity. Genetically-modified rodents have proven to be a powerful tool for the study of aging. For reviews, see [70,71,72,73,74,75,76]. However, the manipulation of some of these genes, although contributing significantly to ameliorating age-related alterations in different tissues, has not had a real impact on longevity itself. It is inherently difficult to determine which of the molecular, cellular, morphological, and functional, or “whole-animal” characteristics represent mechanisms of delayed/accelerated aging and which should be regarded as markers of younger/older biological age. Consequently, although mouse models of accelerated aging may not fully model the natural aging process, they have been employed as alternatives to shed light on the mechanisms underpinning degenerative processes associated with aging. Here, we will address the most relevant animal models that have shown alterations at the testicular level. 

The age-related decline in circulating GH (growth hormone) levels in men is interpreted both as a symptom of neuroendocrine aging (as one of the causes of altered body composition and other unwelcomed symptoms of aging) and as a mechanism of natural protection from cancer and other chronic diseases. Importantly, there is increasing evidence that isolated genetic GH deficiency and GH resistance in humans protects from age-related disease [77,78] and extends healthspan assessed by a variety of criteria [78]. Very interestingly, it was shown early on that mutations in the GH/IGF-1/mTOR (growth hormone/insulin-like growth factor-1/mammalian target of rapamycin) pathway could lead to animals with extended or reduced lifespan. In fact, reduced somatotropic signaling was associated with extended longevity while overexpression of GH was associated with reduced longevity; for reviews, see [79,80]. In this context, GH transgenic (Tg) mice and mice with various progeroid syndromes offer opportunities to study the effects of accelerated aging on reproductive functions [81,82,83]. 

In contrast, association of reduced GH signaling and remarkably extended longevity was subsequently described in mice with hypopituitarism (including GH deficiency), in mice with isolated genetic GH deficiency, and in mice with GH resistance [84,85,86,87]. The Snell dwarf and Ames dwarf mice with mutations in the Pit-1 and Prop-1 genes, respectively, as well as the GH receptor knockout (GHRKO) and the GH releasing hormone knockout (GHRHKO) mice, are classical animal models with delayed aging [83,88,89,90,91,92]. GH-deficient dwarf rats have also been studied [93]. Testicular aging analyses performed on some of these genetically modified rodent models have allowed us to report very clear aging-related alterations in the testis and a negative association between longevity and inflammatory processes, oxidative state, and apoptotic events. Thus, in long-lived (Ames dwarf and GHRHKO) mice, we described the down-regulation of apoptotic germ cell number, macrophage cell number, cyclooxygenase 2 (COX2) expression, prostaglandin D2 (PGD2) production, and lipid peroxidation, [83]. Conversely, mice with decreased longevity (GH-Tg) displayed up-regulation of germ cell apoptosis, macrophage cell number, IL-1β, NLRP3, and COX2 expression, PGD2 production, lipid peroxidation, and catalase expression [83] and unpublished data. GH-Tg mice also display Leydig cell hypertrophy with well-developed cytoplasmic lipid droplets, as well as some seminiferous tubules with arrested spermatogenesis [94], because normal levels of GH promote the generation of proper numbers of mature Leydig cells. The very evident inverse association of COX2 expression and longevity was confirmed with the development of an inducible COX2-Tg mouse model [95] in which post-natal over-expression of COX2 led to short-lived mice that displayed a panel of aging-related phenotypes with increased cellular senescence in virtually every tissue, including the testes. Moreover, COX2-Tg males showed reduced testicular size and number of mature spermatozoa [95]. 

A different, new example of a genetically modified animal model is the senescence-accelerated mouse prone 8 (SAMP8). This mouse strain is exposed to elevated levels of oxidative stress from an early age in various tissues, including the testes [96]. Using SAMP8 and SAMR1 (senescence-resistant inbred strain, the normally aging control group for SAMP strains) mice, Zhao et al. [97] have recently demonstrated that oxidative stress and chronic inflammation are involved in the decline in testosterone production both in vivo and in vitro in aged Leydig cells. Their results highlight the importance of COX2 in the regulation of the age-related decline in testosterone synthesis by providing evidence that activation of two signaling pathways, NF-κB and p38 MAPK, leading to COX2 up-regulation is functionally linked to the oxidative stress response and chronic inflammation commonly observed in aging. In addition, Sprague Dawley rats over-expressing Regucalcin (Rgn, a Ca^2+^-binding protein also known as senescence protein-30) have shown some signs of delayed aging, mainly regarding sperm quality [98]. These include a higher percentage of viable sperm, a lower total amount of oxidant molecules and decreased lipid peroxidation relative to their wild-type littermates [98].

Another illustration of a genetically modified animal model that is not only short-lived, but which also displays signs of premature testicular aging is CDGSH iron sulfur domain 2 (Cisd2)-deficient mice. Cisd2 is a redox active protein localized to the endoplasmic reticulum. It is considered to be relevant for the maintenance of endoplasmic reticulum and mitochondrial structure and function; its expression decreases with age [99]. The alterations that have been reported in Cisd2-deficient mice include reduced Leydig cell and Sertoli cell number, decreased circulating testosterone, increased LH/testosterone ratio, and decreased expression of steroidogenic mRNAs (Lhcgr, Star, Cyp11a1, Hsd3b6, Cyp17a1, Hsd17b3), appropriately modeling primary testicular dysfunction observed in aging men [100].

New evidence is being reported that necroptosis mediates, at least, some of the alterations observed in aged testes. Receptor-interacting protein kinase 3 (RIPK3) is one of the kinases involved in the activation of necroptosis, which is a form of programmed necrotic cell death caused by the tumor necrosis factor family of cytokines [101]. Upon activation, RIPK3 phosphorylates pseudokinase mixed lineage kinase domain-like (MLKL), causing it to form oligomers and translocate to the plasma membrane, where it disrupts membrane integrity, resulting in necrotic cell death. While conducting a study analyzing the impact of necroptosis on the progression of atherosclerosis [102], a group of authors found that the testes of kinase RIPK3 knockout mice looked remarkably young, even at advanced ages. A comprehensive follow-up study [103] revealed that the RIPK3-dependent phosphorylation of MLKL is detected in spermatogonial stem cells in the testes of old, but not young, wild-type mice. They also reported that in aged wild-type mice, the number of cleaved caspase-8 and cleaved caspase-3 positive cells decreased within the seminiferous tubules, but increased in the Leydig cell population. This reduction in caspase-8 may explain how it is that necroptosis, but not apoptosis, occurs in the seminiferous tubules of aged mice.

In addition, mice with genetically Leydig cell-specific impairment of autophagy (Atg7 or Atg5 knockout) display aging-related alterations in the Leydig cell population, such as decreased number of mitochondria, uptake of cholesterol and testosterone synthesis, which ultimately lead to a reduction in serum testosterone levels in these animals [104].

Chemically-accelerated reproductive aging rodent models have also been used in the last few decades, mainly the D-galactose (D-gal)-injected model, which is typically established by administering consecutive subcutaneous D-gal injections to animals for approximately six to eight weeks. Although it has been frequently used for aging research in extra-gonadal tissues, it has not been extensively used for studies of testicular aging. D-gal is a reducing sugar that normally exists in the body. However, when D-gal concentration exceeds normal levels, it is converted to aldehydes and H_2_O_2_ [105]. Studies performed on D-gal or D-gal/NaNO_2_ injected mice or rats have shown decreased testicular weight and volume [106,107], decreased sperm count, increased numbers of immotile and abnormal sperm [106,107,108,109,110,111], reduced serum testosterone levels [106,110,111,112,113], increased apoptotic index [107,110], increased lipid peroxidation [108,110,111,112,113,114], decreased total antioxidant capacity [111,113,114], increased expression of pro-inflammatory cytokines TNF-α, IL-1β and IL-6 [110] and activation of the p19/p53/p21 pathway, which is associated to senescence [110,111].

However, the use of physiologically aged animals is still a very desirable aim for researchers in testicular aging as a way of validating the different alterations found in the testes of genetically modified organisms. In this context, the male Brown-Norway rat is generally used as a model for reproductive aging, given that it does not become obese and it experiences fewer age-related tumors of the endocrine or reproductive system than other rat strains, thus providing a disease-free model for studying male reproductive aging [115]. Testicular morphometry showed age-related reductions in tubule diameter, decreased total sperm count, and smaller Leydig cell volume [115,116,117,118]. With the use of electron microscopy and tracers, disruption of the functional integrity of the blood–testis barrier was reported in this model, along with striking changes in the appearance of Sertoli cells [119]. Microarray and protein analyses revealed lower expression levels of adherens junctions-related proteins, including Jam2, Ocln, cdh2, ctnna, cldn11, and some GTPases (Rac1 and cdc42) involved in the recruitment of cadherins to the adherens junctions [120]. These structural alterations have substantial effects on spermatogenesis. However, perhaps the most significant use of this experimental model has to do with the extensive analysis of testosterone biosynthesis during aging. Interestingly, in the Brown-Norway rat, testosterone levels decrease with age despite unchanging LH and increasing FSH levels, just as was reported in aging men, but without loss of Leydig cells [115,116,117,118,121,122]. Early studies have demonstrated that testicular fragments, as well as Leydig cells purified from aged Brown-Norway rats, exhibit a reduced maximal hCG-stimulated testosterone production compared to those of young adults [123,124]. In this context, multiple defects have been identified in the steroidogenic pathway of aged Leydig cells, including decreased LH-stimulated cAMP production, reduced expression and/or activity of key players in the steroidogenic pathway (Star, Tspo, Cyp11a1, Hsd3b, Cyp17a1, Hsd17b), decreased autophagic activity of Leydig cells, and increased cellular lipofuscin accumulation [125,126,127,128,129,130,131,132,133]. Interestingly, aged Brown-Norway rat Leydig cells showed increased expression of Cox [121,126,133] and decreased testicular expression of antioxidant defenses (Catalase, Sod1, Sod2, Peroxiredoxin1, GSH) [134,135]. 

Sprague Dawley [135,136,137,138] and Wistar rats [130,139,140] have also been used as physiologically aged models by several authors. The effects of aging resulted in decreased sperm count [136,137,138], viability [137], and kinematics [138], reduced testosterone serum levels [139], testicular weight [137], seminiferous tubules size [138], testosterone concentration [137] and expression levels of antioxidant defenses (Gpx4, Prx4, Gstm5, Sirt1) [138], endoplasmic reticulum stress and unfolded protein response proteins (Grp78, Atf6, Atf4, p-Perk, p-Ire1, and Xbp1) as well as increased endoplasmic reticulum stress-related apoptosis proteins expression (Caspase 12, Chop, and Caspase 3) and TUNEL-positive apoptotic germ cells [137]. Aged Leydig cells also showed increased lipid peroxidation, reduced glutathione levels, lower expression levels or catalytic activity of antioxidant enzymes (Sod1, Sod2, Gpx1) [134], and decreased autophagic activity of Leydig cells [130]. Interestingly, autophagy has been reported to be involved in the maintenance of testosterone levels in the rat testis during aging, because treatment with rapamycin, an autophagy activator, enhanced LH-stimulated steroidogenesis in Leydig cells from aged, but not young rats [130]. 

Naturally aged mice (e.g., C57BL/6, Swiss mice) have also been employed in testicular aging studies, showing decreased serum testosterone levels alongside signs of increased testicular inflammation (higher levels of IL-1β and IL-6) and interstitial senescence (i.e., up-regulation of p53, p21, p16, and TGF-β expression and increased nuclear translocation of transcription factor FOXO4 in aged Leydig cells) [141]. Age-related changes in the expression levels of key steroidogenic components (decreased Star, Cyp11a1, Cyp17a1, and Hsd17b1), endoplasmic reticulum stress markers (increased Grp78 and Chop), and antioxidant defenses (decreased Sod2, Gpx4, and Sirt1) were reported in testicular tissue [142]. Because knocking out Nrf2, a master regulator of phase 2 antioxidant genes, further reduces serum testosterone levels [143], these results support the hypothesis that, over time, increases in oxidative stress contribute to, or cause, the reduced testosterone production that characterizes aged Leydig cells. Some authors have also, reported increased apoptotic events [103] and ROS levels [144] in aged mouse Leydig cells. In addition, an increased number of testicular macrophages were reported [138] and the typical interdigitations between testicular macrophages and Leydig cells were not found in the testis of aged mice [145]. Additionally, lipofuscin granules, like those found in Leydig cells from aging mice, were observed in the cytoplasm of testicular macrophages [145]. Because of their intense oxygen metabolism and being an important source of ROS production, mitochondria have been frequently implicated in lipofuscinogenesis. However, although mitochondria of murine aging testicular macrophages progressively diminished in number and accumulated lipofuscin granules, they generally preserved normal morphology [145]. Since the appropriate microenvironment, formed by macrophages and other interstitial components, is necessary for Leydig cell steroidogenesis [132], increased number of macrophages in the testes of aged animals might result in the disruption of Leydig cell endocrine function. 

Lately, other naturally-aged experimental models have been employed in the studies of testicular function. These included non-human primates, the rhesus macaque [73,146,147] and the common marmoset [73,148,149], as well as alternative rodent species, such as naked mole-rats [150,151] and Syrian hamsters [152]. However, studies that focused mainly on age-related alterations in the testes of these experimental models are either scarce or non-existent. For instance, the naked mole-rat has an extraordinary maximum lifespan of 30 years, showing very few age-associated deficits and maintaining high locomotory activity, heart health, bone quality, and reproductive capacity for about 75% of their lifespan [150,151,153]. Although interest in naked mole-rats has increased dramatically over recent years because of their many unusual traits, none of the reports have addressed testicular aging per se; they have focused on the differences between the breeder and non-breeder males of this species [153,154,155]. 

Non-human primate models are of particular interest for pre-clinical testing due to their close evolutionary history with humans. Old World apes such as chimpanzees are very long-lived, with animals in captivity living up to 60 years, making it difficult to employ them in aging-related research. Additionally, they have been classified as endangered species by the International Union for Conservation of Nature (IUCN). Thus, although they are the closest living relatives of humans, research using chimpanzees has virtually stopped. New World monkeys such as the common marmoset (*Callithrix jacchus*) are the shortest-lived anthropoid primates. With an average lifespan of 5 to 7 years and a maximum lifespan of 16.5 years, they have become a standard non-human primate aging model [147] and have been used in some interesting studies. Regarding testicular aging, testosterone has been found to decrease in aging male marmosets, but they remain capable of reproduction, and no known phenotypic changes are associated with the decreased testosterone [147,156]. More recently, using a mass spectrometry-based proteomics approach, a comprehensive analysis of age-related alterations in the testicular proteome of the common marmoset has been reported [157]. Findings included an increase in the levels of anti-proliferative proteins (e.g., Cdkn2a, Prelp, and Ogn) and actin-binding proteins that inhibit contractility and thus presumably affect the function of testicular peritubular cells (namely, Cnn1, Cald1, and tropomyosins). Moreover, immunostaining studies have revealed increased collagen deposition in the testes of old animals, which is associated with fibrosis. By isolating testicular peritubular cells from young *Callithrix jacchus* testes and culturing them for a low (2–3) or high (>10) number of passages, an in vitro model of cellular senescence was established. An in-depth proteome analysis of these cell cultures revealed that senescent testicular peritubular cells had lower expression levels of proteins involved in smooth muscle activity (mainly, Cnn1, Acta2, Myh11, and desmin). In addition, the secretome analysis showed decreased abundance of several proteins (e.g., fibronectin, laminins, collagens, peroxiredoxin 4, superoxide dismutase 1 and 2) [158].

The Syrian hamster (*Mesocricetus auratus*) is one of the most widely used rodents for biomedical research, representing 13% of total laboratory animals used to investigate human diseases [159]. Because of the phylogenetic similarities that hamsters and humans share, Syrian hamsters have several advantages over other rodents and have therefore become the preferred model for the study of several human diseases, such as cancer, atrial thrombosis, epilepsy, muscular dystrophy, periodontal, pancreatic and inflammatory diseases [159]. However, there were very few studies of testicular aging in this species. Our group has described that, although body weight, testicular weight, testicular volume, and testicular density remained unchanged throughout the aging process in our hamster colony, we detected a significantly higher number of seminiferous tubules in aged hamsters (18–22 month-old) than in young adult animals (5 month-old) [152]. Mukherjee and Haldar [160], however, did detect significantly reduced testicular weight in aged hamsters (24 month-old). Using Picrosirius red staining and polarization microscopy we were able to showcase the increased fibrotic thickening of the tubular wall and the higher level of disorganization of the collagen-containing tubular wall in aged hamsters [152]. Classic inflammatory markers were significantly increased in testes of aged hamsters in comparison to young animals. They included IL-1β, NLRP3, and COX2 expression, as well as PGD2 production and testicular macrophage numbers [152]. Regarding the redox status of aged Syrian hamster testes, our group reported an age-dependent increase in testicular lipid peroxidation and antioxidant enzyme catalase expression [152], along with a marked decrease in testicular concentration and content of the naturally occurring non-enzymatic antioxidant melatonin [152]. Mukherjee and Haldar, [160] also reported lower testicular melatonin concentration and higher levels of lipid peroxidation in aged hamsters; however, the activity of antioxidant enzymes (Sod, Catalase, Gpx) was decreased in their animals. They also showed the age-related decrease in plasma testosterone levels and testicular expression of steroidogenic components (Star and Cyp11a1) and the subtype 1 melatonin receptor. In a different study, no histological differences were detected between the interstitial tissue of young and old hamsters unless transmission electron microscopy was used. In aged animals, Leydig cells exhibited signs of cellular degeneration, with fewer mitochondria, a greater number of lysosomes, a strongly vacuolated cytoplasm, and numerous clumps of chromatin, while macrophages were characterized by numerous cytoplasmic extensions and slightly kidney-shaped heterochromatic nucleus. However, Leydig cells and macrophages maintained contact through cytoplasmic projections from the Leydig cells located in cytoplasmic invaginations of the macrophages [161].

## 3. Interventions to Reverse Aging-Related Testicular Alterations

**Figure 2:**
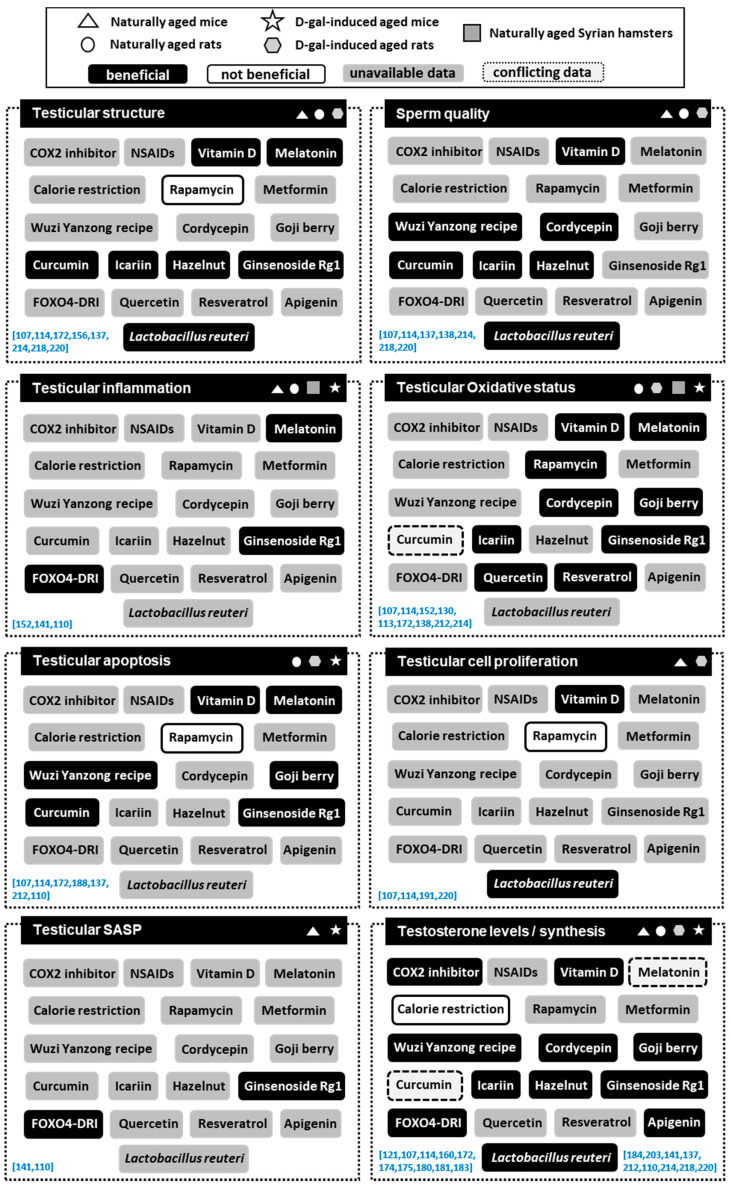
Effects of main anti-aging interventions on testicular aging in different experimental animals. Main anti-aging interventions were categorized into beneficial or not beneficial according to their ability to reverse aging-related testicular alterations. Conflicting results or lack of data on the different interventions were also included. Corresponding references are listed between brackets (blue).

There have been a number of reports on the beneficial effects of different interventions on the structure and/or function of the human testis, but mostly in pathological conditions such as testicular torsion, ischemia/reperfusion, diabetes, and idiopathic infertility, or by following the exposure to various toxic agents (e.g., cadmium, cyclophosphamide, busulfan, methotrexate, liponavir, bisphenol A or microwave radiations). To our knowledge, there is no available information about successful interventions that have been implemented in elderly men to improve testicular function. For this reason, this review will only focus on those reports that have addressed some aspects of testicular aging using either naturally, chemically-induced or, genetically-accelerated aged animals (Figure 2).

### 3.1. Common Anti-Inflammatory Drugs

We failed to find information regarding potential actions of steroidal drugs (e.g., prednisone, cortisone, and methylprednisolone) on the aging testis. In contrast, some studies have investigated the impact of nonsteroidal anti-inflammatory drugs (NSAIDs) in cellular and animal models of testicular aging. Incubation of aged Leydig cells in the presence of the COX2 inhibitor NS398 led to a significant increase in testosterone production [121]. Furthermore, in aged rats fed the COX2 inhibitor DFU [5,5-dimethyl-3-(3-fluorophenyl)-4-(4-methylsulphonyl)phenyl-2(5H)-furanone], blood testosterone concentrations and testicular StAR expression levels increased over those found in rats receiving no DFU [121]. These results were expected, given that aged testes and aged Leydig cells express higher levels of COX2 [83,121,126,133,152] and that PGF2α was reported to inhibit hCG-induced testosterone production in hamster Leydig cells [162,163]. Although the impact of other NSAIDs such as indomethacin, paracetamol, aspirin, and ibuprofen on testes is currently being studied in young and middle-aged men, no data on the impact of these drugs on testicular aging are available at present. It is discouraging that all four of these mild analgesics were shown to cause multiple endocrine disturbances in organo-cultured adult human testis [164,165,166] and human testicular peritubular cells [167].

### 3.2. Common Antioxidant Compounds

Several studies have indicated that common oral antioxidants (e.g., vitamin C, vitamin E, vitamin D, selenium, folate, zine, and carnitine) improve sperm quality (i.e., sperm count, motility, and morphology) both in men and in animal models reviewed by [168,169]. However, these studies have been focused on infertility-related sperm characteristics in young, rather than older, men. In addition, individual studies investigating the therapeutic effect of such products in aged experimental animals are scarce. 

With age, renal synthesis of 1,25-dihydroxy-vitamin D3 or calcitriol (the active form of vitamin D) declines. Vitamin D supplementation has been found to alleviate the manifestations of male reproductive aging. Jeremy et al. [107,114] subcutaneously administered vitamin D (40–400 UI/kg) twice a week for 6 weeks to D-gal-induced aged rats. Their studies show that vitamin D supplementation resulted in a decrease in apoptotic germ cells, and an increase in proliferating germ cells. Vitamin D also significantly decreased testicular peroxidation and increased the activities of antioxidant enzymes Sod and catalase [107]. In addition, the administration of vitamin D to D-gal-induced aged rats produced significant improvements in sperm parameters, testicular histology, and serum testosterone levels [114].

Melatonin exerts antioxidant actions in many, and likely all, species. It has been proposed to have evolved as an antioxidant to protect organisms from the increasing concentration of oxygen in the environment and the free radicals that naturally ensued. Melatonin is also known to have anti-inflammatory, anti-apoptotic, and anti-cancer actions, reviewed by [170]. Melatonin is a neurohormone that is produced primarily by the pineal gland; however, it can reach the testis, pass through the blood–testis barrier and directly modulate testicular activity [171]. Testicular melatonin levels show an age-dependent decrease at least in Syrian hamsters [152,160]. To the best of our knowledge, our studies [152] and those of Muratoğlu et al. [172] and Mukherjee and Haldar [160] were the only ones that addressed the potential effects of melatonin on testicular histology/function in naturally aged animal models. 

Muratoğlu et al. [172] used aged Wistar albino rats that were subcutaneously injected (in the afternoon) with either melatonin (10 mg/kg) or saline over a period of 21 days. In their study, the authors reported that melatonin partially reverted the characteristic atrophic lesions of aged testes, increased the germinal epithelium height and reduced the number of TUNEL-positive apoptotic cells compared to non-treated aged rats. In contrast, melatonin administration did not reverse the aging-related increase in lipid peroxidation, an unexpected result considering the unequivocally antioxidant properties of melatonin. The authors used a dose of melatonin based on previous studies from Akbulut et al. [173] in which melatonin seemed to have some antioxidant effect on the gastric mucosal tissue of aged rats. However, the time of melatonin administration is crucial, at least for studies involving steroidogenic-related alterations. In this context, no significant changes in the levels of serum testosterone have been recorded in melatonin-treated aged rats by this group [172]. This outcome was unexpected because melatonin was shown to dose-dependently inhibit hCG-induced testosterone production in vitro [174,175], and afternoon melatonin injections, even a single dose, were reported to inhibit testicular steroidogenesis in rats [176]. Unexpectedly, aged Syrian hamsters that received a daily morning (10 a.m.) intraperitoneal 10 mg/kg dose of melatonin at 10:00 a.m. for 7 days did experience a significant increase in plasma testosterone levels and testicular Star expression. However, in this study, testicular melatonin concentration did not increase following intraperitoneal melatonin administration, suggesting that some of the beneficial effects of melatonin administration might not be due to the actions of melatonin itself [160].

As stated before, the intervention methods for assessing the possible beneficial effects of melatonin in testicular aging have been subcutaneous or intraperitoneal injections. This approach may not have been optimal because it can lead to potential adverse effects on animal well-being and because the final testicular melatonin levels are uncertain. Both of these negative aspects can affect subsequent results. In view of these limitations, we have decided to use a unique approach based on a distinctive characteristic of Syrian hamsters which respond to a simple modulation of photoperiod by an increase in testicular melatonin levels. By transferring aged Syrian hamsters from a long-day (LD, 14 h light per day) to a short-day (SD, 6 h light per day) photoperiod for 16 weeks, we ensure that testicular melatonin increases, with concentrations remaining within a physiological range, thus minimizing the potential for detrimental effects [152]. In fact, testicular melatonin concentration in aged hamsters that had been maintained in a SD-photoperiod for 16 weeks was completely restored to the levels in LD-young animals, or even slightly surpassed these levels. Under these conditions, we found crucial improvements in testicular overall status in SD-aged hamsters compared to LD-aged hamsters, including a reduced expression of inflammatory markers (IL-1β, NLRP3, and COX2 expression, as well as PGD2 production and testicular macrophage number) and a decreased pro-oxidant environment (decreased testicular lipid peroxidation and elevated expression of the antioxidant enzyme Catalase) [152]. Supporting these results, the numbers of apoptotic germ cells and macrophages, as well as COX2 expression, PGD2 production and lipid peroxidation, were also reduced in testes of long-lived Ames dwarf and GHRHKO mice accompanied by an increased testicular melatonin concentration [83] and unpublished data. 

In addition to its antioxidant actions, studies on brain function have shown that melatonin exerts a dual effect, activating autophagy under age-related neurodegenerative and neurodestructive conditions, but blocking autophagy under specific pathological conditions (i.e., exposure to neurotoxic agents such as methamphetamines and kainic acid, ischemia, and reperfusion) [177]. A recent study by Wang et al. [178] suggested that melatonin regulates the crosstalk between autophagy and apoptosis via the activation of SIRT3 in Leydig cells resulting in ameliorated testicular injury. However, this study was not performed in aged animals. Mice with genetically Leydig cell-specific impairment of autophagy display aging-related alterations which ultimately lead to a reduction in serum testosterone levels [104]. In summary, it appears that the potential of melatonin to influence testicular aging, especially to target autophagic flux, has not yet been fully explored.

### 3.3. Nutrient-Sensing Pathway Inhibition

The use of calorie restriction interventions has proven to be a useful tool to extend lifespan and delay the onset of age-related diseases in numerous organisms ranging from yeast to mammals reviewed by [179]. However, a negative effect of calorie restriction on reproduction has been documented, especially when the degree of restriction is more severe. For example, food intake restrictions of 40–50% caused a reduction of mean, maximal, and basal LH levels and LH pulse amplitude in rats [180] and reduced testicular weight and plasma testosterone levels in white-footed mice [181]. In contrast, mild calorie restriction (20%) in long-lived GHR knockout mice and short-lived GH-Tg mice did not have any effects on testicular testosterone content or expression of Fshr, Ar, Hsdd3, Cyp17a1 [182]. In addition, adult-onset mild calorie restriction (10–20%) for 12 months in wild-type mice did not significantly impact plasma testosterone levels or testicular weight and testosterone concentration [183]. In studies performed on Leydig cells isolated from aged Brown-Norway rats that had been maintained under a 40% level of calorie restriction, Chen et al. [184] reported that almost all of the major changes in gene expression (genes related to cholesterol metabolism and steroidogenic enzymes) that were seen in aged-Leydig cells were not reversed by short-term or long-term exposure to caloric restriction. Overall, despite the documented benefits for health and lifespan, only mild calorie restriction is, apparently, not deleterious to testosterone production and this intervention does not seem to have a clear beneficial effect on testicular aging. 

Rapamycin is a well-known inhibitor of mammalian target of rapamycin (mTOR) nutrient signaling pathway and inducer of autophagy [185]. It is considered a calorie restriction mimetic. Regarding testicular function, the mTOR pathway is associated with cell-specific effects contributing to testosterone production by Leydig cells, Sertoli cell proliferation, redox balance, and metabolic activity, Sertoli cell–blood-testis barrier dynamics, and proliferation/apoptosis of germ cells reviewed by [186]. Rapamycin administration extends murine lifespan, reflecting its anti-aging properties [187,188,189,190]. In the testis, however, rapamycin does not seem to have anti-aging effects. In fact, in vivo studies performed in rats and mice showed that rapamycin increased testicular degeneration following an orderly loss of all stages of spermatogenesis from the most mature to least differentiated cells (i.e., spermatids, spermatocytes, differentiating spermatogonia, and primary spermatogonia) [188] due to inhibition of proliferation [191] and differentiation [192] of spermatogonia. There is an ongoing long-term study to test the effect of a daily dose of rapamycin on longevity and healthy aging in a cohort of middle-aged marmosets (mixed sexes) [193]. The results of this work should indicate whether rapamycin will affect the testes of this non-human primate species similarly to its effects in laboratory rodents. Different results were seen in in vitro conditions by Li et al. [130]. In Leydig cells isolated from young and aged Sprague Dawley rats, enhancement of autophagic activity with rapamycin was associated with antioxidant properties (decreased ROS levels) in aged rat Leydig cells, but not in young cells.

Metformin (dimethyl biguanide) is a synthetic derivative of guanidine, isolated from the extracts of *Galega officinalis*. Metformin is an oral anti-diabetic drug that has been recommended as the first-line therapy for patients with type 2 diabetes mellitus [194]. Like rapamycin, metformin has been described as a calorie restriction mimetic. It is also an mTOR inhibitor and an inducer of autophagy, although indirectly so and via multiple mechanisms [179]. In animal models, multiple beneficial effects of metformin have been reported across species with the magnitude of these benefits varying with the dosage, sex, and age at onset of treatment. However, longevity was extended by metformin administration only in *Caenorhabditis elegans*, reviewed by [195], and in only one of the tested strains of mice [196]. An extensive review by Tseng [197] summarized the effects of metformin on male reproductive health, specifically on erectile dysfunction, steroidogenesis, and spermatogenesis (showing beneficial effects on all issues, mostly in animal models). However, none of the studies were performed in aged animals or aged men. We can only conclude that not enough research on this topic has been published; thus, there are many opportunities to add to the current resources of anti-aging therapies in testicular aging. 

Collectively, the results of the interventions that inhibit nutrient-sensing pathway are somewhat counterintuitive and remind us that particular attention has to be given to identifying testicular responses to any anti-aging therapy that was developed on the basis of its effects on extra-gonadal tissues.

### 3.4. Senolytics and Senomorphics

One of the hallmarks of aging is an increased number of senescent cells which secrete a variety of bioactive factors called senescence-associated secretory phenotype (SASP) [198,199]. Targeting senescent cells has recently emerged as a therapeutic target for treating age-related diseases. In this context, senolytics are compounds that selectively and actively eliminate senescent cells by inducing apoptosis, whereas senomorphics are compounds that suppress SASP by targeting pathways such as p38 MAPK, NF-κB, IL-1α, mTOR, and PI3K/AKT, without inducing apoptosis. Some of these compounds have been classified as calorie restriction mimetics as well [179]. 

The first generation of senolytic drugs include different compounds such as Dasatinib, Quercetin, Fisetin, Navitoclax, Curcumin, Luteolin, and FOXO4-related peptide, among others [199]. Many of these are naturally occurring flavonoids, once known as vitamin P, commonly found in various types of herbs, vegetables, and fruits. We will only refer to those compounds that have been employed to assess potential beneficial effects on testicular senescence/aging. On the other hand, senomorphics include polyphenols such as Resveratrol and Apigenin, Metformin, Cortisol/Corticosterone, Wogonin, Kaempferol, and NDGA [200]. The beneficial actions of many of these plyphnenols on testicular steroidogenesis have been reviewed by Martin and Touaibia [201].

In a recent study, Hamza et al. [113] have developed a D-gal-induced aging mouse model. Using this model, the authors showed that daily intraperitoneal administration of Quercetin (20 mg/kg) or Resveratrol (20 mg/kg) for 30 days significantly decreased testicular lipid peroxidation and increased antioxidant enzymes activities (Catalase, Glutathione reductase). In aged Leydig cells, there was also an up-regulation of Cox2 expression and a reduction in StAR gene expression and testosterone production [121]. However, incubation of aged Leydig cells in the presence of a Cox2 inhibitor can restore testosterone production. Interestingly, flavonoids such as Luteolin [202], and Apigenin [203] can promote StAR expression and steroidogenesis by inhibiting Cox2-dependent signaling in in vitro cell cultures. In addition, beneficial effects of Resveratrol following the exposure to various toxic agents has been reviewed by Pasquariello et al. [204] and are beyond the scope of this review since these reports were not conducted in aging experimental animals.

Curcumin is a bioactive substance found in turmeric and that has strong antioxidant attributes mostly related to its free radical scavenger properties [205]. Muratoğlu et al. [172] have investigated the effects of intraperitoneal administration of curcumin (6 and 30 mg/kg) over a period of 21 days in the testis of aged Wistar albino rats. They reported a general improvement of testicular histology describing uniform seminiferous tubules with normal interstitial histology, increased germinal epithelium height, and a decreased number of TUNEL-positive apoptotic cells compared to non-treated aged rats. Overall, these general testicular improvements resulting from curcumin administration were not accompanied by beneficial effects on plasma testosterone levels. Moreover, the observed increase in testicular antioxidant defenses, such as GSH concentration, was not sufficient to reverse the lipid peroxidation found in aged testes [172]. The beneficial effects of curcumin following the exposure to various toxic agents has been reviewed by Martins et al. [206], and are beyond the scope of this review.

FOXO4 (Forkhead box O4) is a transcription factor that maintains senescent cell viability by targeting p53 to the nucleus and preventing it from inducing apoptosis senescence-associated genes, such as p53. FOXO4-DRI is a peptide antagonist designed to block the interaction of FOXO4 and p53 [207], thus causing p53 to be excluded from the nucleus and directed to mitochondria for induction of apoptosis, ultimately eliminating the senescent cells. In a recent study of Zhang et al. [141] FOXO4-DRI treatment of naturally aged C57BL/6 mice alleviated testicular senescence phenotype by decreasing the expression levels of senescence-associated proteins (p53, p21, p16, and TGF-β) and inflammatory markers (IL-1β and IL-6) and by improving serum testosterone levels. This intervention might be of relevance for humans given that in human testes FOXO4 was specifically expressed in Leydig cells [141]. Even though no significant difference in FOXO4 protein levels was detected between testes from young and old men, a clear increase in FOXO4 nuclear translocation was found in Leydig cells in old men. These findings suggest the potential efficacy of FOXO4-DRI for the treatment of the age-associated male late onset hypogonadism [141]. 

### 3.5. Herbs and Nutraceuticals from Traditional Oriental Medicine

A growing number of reports have demonstrated that some prescriptions of Traditional Chinese Medicine could play distinct roles in anti-aging therapy. Chinese compound prescriptions are usually composed of several kinds of herbs, each of which contain multiple active constituents. Traditional Chinese Medicine not only involves multiple bioactive components with various pharmacological activities, but might also generate other bioactive (or inactive) metabolites when delivered in vivo. Hence, it is difficult to determine whether the anti-aging effect is due to the synergistic therapeutic efficacies or to a single mechanism. Some authors have employed Traditional Chinese Medicine recipes while others have attempted to use single components. We will attempt to summarize those reports that have addressed possible anti-inflammatory, anti-oxidative, and/or anti-apoptotic properties in naturally, chemically- or genetically-induced aged animals. 

Wuzi Yanzong recipe is a classical Traditional Chinese Medicine prescription comprised of five components: Plantaginis semen, Rubi fructus, Schisandrae chinensis fructus, Lycii fructus, and Cuscutae semen. Treatment of 18 month-old Sprague Dawley rats with Wuzi Yanzong recipe for 4 months significantly increased sperm count and viability, testicular weight, and testosterone concentration, while decreasing testicular concentration of estradiol. Furthermore, the Wuzi Yanzong recipe significantly decreased the number of TUNEL-positive cells, possibly by up-regulating the expression levels of endoplasmic reticulum stress-responsive proteins (Grp78, p-Perk, Atf4, p-Ire-1α, Xbp1, and Atf6), and down-regulating the expression levels of pro-apoptotic proteins (p-Jnk, Caspase 12 and Chop) in testicular germ cells [137].

*Cordyceps militaris* Linn. is a valuable edible mushroom used extensively as a crude medicament and food in Asian countries. Among the components of *C. militaris*, cordycepin, also known as 3-deoxyadenosine, a purine nucleoside derivative, is a well-studied active constituent with significant biological properties (i.e., anti-tumor, anti-viral, anti-inflammatory, and anti-atherosclerotic effects) [208]. Because of its structural similarity to adenosine, certain enzymes cannot discriminate between the two molecules. Therefore, cordycepin is readily phosphorylated intracellularly, enabling its participation in several physiological and biochemical reactions. With an oral administration of a daily dose of 20 mg/kg of cordycepin, Kopalli et al. [138] observed many improvements in aged Sprague Dawley rats including increased testicular expression levels of genes involved in antioxidant defenses (Gpx4, Prx4, Gstm5, and Sirt1) and hormone receptors genes (Ar, Fshr, Lhr) as well as improvement in sperm quality (motility and progressiveness).

Goji berry is a herbal medicine widely used in Asian countries. It is the fruit of *Lycium chinense* P. Mill., commonly referred to as a “superfood” because of its nutritive and antioxidant properties; it has become very popular over the last decade. It contains various compounds such as betaine, β-sitosterol, scopoletin, β-carotenes, phenolic compounds, and polysaccharides [209,210,211]. Recently, Jeong et al. [212] used intragastric gavage to administer goji berry (150–300 mg/kg/day) to aged Sprague Dawley rats for six weeks. The authors found that goji berry improved serum testosterone levels while decreasing testicular apoptosis (reduced Bax/Bcl-2 ratio) and minimizing oxidative damage (decreased 8-OHdG levels and increased antioxidant enzyme Sod activity). 

Ginsenosides are the main active ingredients of Panax ginseng. Studies have shown that ginsenosides such as Rg1 display many pharmacological effects, such as relieving fatigue, improving immunity, slowing aging, inhibiting metastasis of cancer cells, and regulating blood glucose [211]. Studies performed on D-gal-injected chemically-induced aging mice showed that daily intraperitoneal injections of Rg1 (20 mg/kg) for 28 days reduced aging-related decline in serum testosterone levels and improved overall testicular appearance by reducing spermatocyte apoptosis and the number of senescent cells (the latter possibly by down-regulation of p53, p21, and p19 expression) [110]. Regarding the oxidative status of the testis, lower lipid peroxidation and higher total antioxidant capacity followed ginsenoside Rg1 administration. Moreover, Rg1 was able to reduce testicular expression levels of inflammatory markers (TNF-α, IL-1β, and IL-6) in aged-mice [110]. 

Icariin is a flavonoid isolated from *Herba epimedii*, a traditional Chinese and Korean herbal medicine and it is considered to be the main active component in this plant. Makarova et al. [213] reported that erectile dysfunction was reversed by oral administration of icariin to aged rats. Studies performed by Zhao et al. [214] on aged Sprague Dawley male rats revealed that icariin (100–200 mg/kg) effectively ameliorated the age-related decline in testicular function by increasing testicular and epididymal weights and indices, sperm count, sperm viability, testicular testosterone concentrations, the seminiferous tubule diameters and the height of the seminiferous epithelium. The potential use of icariin to promote testosterone secretion was reported also in earlier studies [215,216]. Although not focused on testicular aging, Chen et al. [216] have reported that icariin can induce testicular expression of steroidogenesis-related genes (Lhr, Star, Cyp11a1, Cyp17a1, and 3β-HSD1) which could explain the beneficial effect observed by Zhao et al. [214]. These authors also reported an increase in antioxidant enzyme Sod activity and a decrease of testicular lipid peroxidation, but these beneficial effects were dose-dependent (50–100 mg/kg). When the dose of icariin was increased to 200 mg/kg, lipid peroxidation was increased above the levels measured in non-treated aged rats.

Other flavonoid-enriched plant extracts or herbs have also shown antioxidant properties. These include herba euphorbiae humifusae, the dried whole plant of *Euphorbia humifusa* Willd. Administration of this preparation to D-galactose-induced aged mice improved testicular Sod activity and decreased lipid peroxidation [217].

The origins of hazelnut (also known as filbert and cobnut) can be traced to Asia and Europe. In Traditional Chinese Medicine, hazelnut is known for its ability to tonify blood and qi (also ki or ch’i in Wade–Giles Romanization, is believed to be a vital force forming part of any living entity). The effects of a hazelnut supplemented diet on the reproductive system of young and old male rats were investigated by Kara et al. [218]. Supplementing Sprague Dawley rats’ food with hazelnut (3 g hazelnut/kg body weight) for 30 days improved testicular histopathological variables, sperm quality, seminal plasma and plasma oxidative stress as well as seminal plasma vitamin E, and plasma testosterone levels in both young and old male rats.

### 3.6. Probiotics, Prebiotics, and Synbiotics

Probiotics are live microorganisms that, when administered in adequate amounts, confer a health benefit to the host. The term prebiotic is defined as a substrate that is selectively utilized by host microorganisms, conferring a health benefit. Combining both terms, the denomination ‘synbiotics’ arose, which represents a mixture comprising live microorganisms and substrate(s) selectively utilized by host microorganisms that confers a health benefit on the host. Elderly people, in general, suffer from changes in the gut microbiota composition, leading to a gradual shift toward a reduced bacterial diversity. This implies a decline in valuable microorganisms that exert beneficial health effects, such as protection against pathogens and presenting anti-inflammatory properties [219]. Some very interesting reports have been published on the antioxidant and anti-inflammatory properties of different synbiotics in naturally (C57BL/6 mice), genetically (SAMP8 mice), or chemically-induced (d-gal-induced Sprague Dawley rats or C57BL/6 mice) aged rodent models. No indications for effects at the testicular level were reported in these studies, reviewed in [219]. There is, however a report from Poutahidis et al. [220] in which aged Swiss mice on a normal diet were given oral *Lactobacillus reuteri* supplementation. Testes from *Lactobacillus reuteri*-fed mice were larger compared to aged control mice. These mice also displayed increased Leydig cell number and size, increased serum testosterone levels, and higher sperm concentration. These findings suggest that probiotic organisms may offer practical options for the management of male reproductive disorders frequently associated with aging.

## 4. Concluding Remarks

In-depth understanding of the mechanisms of aging and senescence is one of the key elements of modern cell biology research. To be able to answer such questions as: How is the aging process initiated? What are its stages? and What are their consequences? will facilitate the design of strategies that can slow down these processes. 

Collectively, data generated by our group and by other investigators indicate that a key hallmark of the testicular aging process is chronic low-grade inflammation, named inflammaging. Inflammaging can be triggered by diverse stimuli, such as the accumulation of senescent cells or dysfunction of the immune system. In such a model, the sustained presence of senescent cells over time is associated with the secretion of numerous pro-inflammatory cytokines, growth factors, and matrix remodeling enzymes that impair proper tissue function and promote the aging process. However, in some processes, such as wound healing and liver fibrosis, the elimination of senescent cells could be detrimental; thus, more work will be needed to develop reliable anti-aging therapies based on targeting senescent cells with senolytics and/or senomorphics. Additionally, according to the oxidation–inflammation theory of aging, there is an underlining interdependence between oxidative stress and the occurrence of inflammatory processes. In this regard, results from our group and from others have also made clear that an imbalanced redox status (leading towards a pro-oxidant microenvironment) is another hallmark of testicular aging. Therefore, compounds with antioxidant properties (many of which have no deleterious or cytotoxic effects and are present in many foods or herbs) might be excellent candidates to reduce, if not eliminate, the important effects of aging-related testicular oxidative stress. Non-invasive delivery is another potential advantage of these compounds.

After reviewing the current literature, we conclude that there is a lack of research on the effects of different commonly accepted anti-inflammatory and anti-oxidative therapies or interventions on testicular aging. Because no single therapy is likely to produce all the desired beneficial effects, the combination of different approaches might result in better outcomes. In this context, more research on animal models is needed to identify the combinations that might have synergistic effects. Lastly, translation of animal model-based results to possible anti-aging therapies in elderly men is difficult because of the extremely limited availability of testicular tissue from healthy aging men that could be used for research. Therefore, the possible success of such therapies will have to rely on more readily available data such as restoration of plasma testosterone levels and reversal of androgen deficiency-related alterations in aging men (low libido, erectile dysfunction, poor semen quality, low energy, sleep disturbance, depressed mood, impaired cognition, osteoporosis, and loss of muscle mass or increased body mass index). Although reproductive aging does not necessarily include male infertility, it promotes the development and progression of several comorbidities. Consequently, if successful, anti-aging therapies will not only exert direct effects on testicular physiology, but will also enhance overall health and quality of life in aging men.
